# Circulating MicroRNAs and myocardial involvement severity in chronic Chagas cardiomyopathy

**DOI:** 10.3389/fcimb.2022.922189

**Published:** 2022-08-08

**Authors:** Sergio Alejandro Gómez-Ochoa, Paula Katherine Bautista-Niño, Lyda Z. Rojas, Lukas Hunziker, Taulant Muka, Luis E. Echeverría

**Affiliations:** ^1^ Institute of Social and Preventive Medicine (ISPM), University of Bern, Bern, Switzerland; ^2^ Research Center, Fundación Cardiovascular de Colombia, Floridablanca, Colombia; ^3^ Research Group and Development of Nursing Knowledge (GIDCEN-FCV), Research Center, Fundación Cardiovascular de Colombia, Floridablanca, Colombia; ^4^ Department of Cardiology, Bern University Hospital, Bern, Switzerland; ^5^ Heart Failure and Heart Transplant Clinic, Fundación Cardiovascular de Colombia, Floridablanca, Colombia

**Keywords:** chagas disease, chronic chagas cardiomyopathy, microRNAs, echocardiography, omics

## Abstract

**Background:**

Chronic Chagas Cardiomyopathy (CCM) is characterized by a unique pathophysiology in which inflammatory, microvascular and neuroendocrine processes coalesce in the development of one of the most severe cardiomyopathies affecting humans. Despite significant advances in understanding the molecular mechanisms involved in this disease, scarce information is available regarding microRNAs and clinical parameters of disease severity. We aimed to evaluate the association between circulating levels of six microRNAs with markers of myocardial injury and prognosis in this population.

**Methods:**

Patients with CCM and reduced ejection fraction were included in a prospective exploratory cohort study. We assessed the association of natural log-transformed values of six circulating microRNAs (miR-34a-5p, miR-208a-5p, miR-185-5p, miR-223-5p, let-7d-5p, and miR-454-5p) with NT-proBNP levels and echocardiographic variables using linear regression models adjusted for potential confounders. By using Cox Proportional Hazard models, we examined whether levels of microRNAs could predict a composite outcome (CO), including all-cause mortality, cardiac transplantation, and implantation of a left ventricular assist device (LVAD). Finally, for mRNAs showing significant associations, we predicted the target genes and performed pathway analyses using Targetscan and Reactome Pathway Browser.

**Results:**

Seventy-four patients were included (59% males, median age: 64 years). After adjustment for age, sex, body mass index, and heart failure medications, only increasing miR-223-5p relative expression levels were significantly associated with better myocardial function markers, including left atrium area (Coef. -10.2; 95% CI -16.35; -4.09), end-systolic (Coef. -45.3; 95% CI -74.06; -16.61) and end-diastolic volumes (Coef. -46.1; 95% CI -81.99; -10.26) of the left ventricle. Moreover, we observed that higher miR-223-5p levels were associated with better left-ventricle ejection fraction and lower NT-proBNP levels. No associations were observed between the six microRNAs and the composite outcome. A total of 123 target genes for miR-223-5p were obtained. From these, several target pathways mainly related to signaling by receptor tyrosine kinases were identified.

**Conclusions:**

The present study found an association between miR-223-5p and clinical parameters of CCM, with signaling pathways related to receptor tyrosine kinases as a potential mechanism linking low levels of miR-223-5p with CCM worsening.

## Introduction

Chagas Disease (CD), a tropical neglected infectious disease caused by the protozoan parasite *Trypanosoma cruzi* (T. cruzi), is nowadays considered the parasitic disease with the highest attributable morbidity and mortality burden worldwide (Lee et al., 2013; Bestetti, 2016; Conners et al., 2016; Antinori et al., 2017; Savarese and Lund, 2017; Echeverria and Morillo, 2019; Bern et al., 2019). This burden derives mainly from the consequences of the cardiac form of the disease, known as Chronic Chagas Cardiomyopathy (CCM). CCM is characterized by a higher mortality risk than other etiologies of heart failure (Nunes et al., 2018). CCM represents a unique form of myocardial involvement, distinguished by an extensive replacement of the myocardium by fibrosis, mainly in the inferolateral segments of the left ventricle, which develops as a result of the chronic inflammatory response secondary to *T. cruzi* persistence in blood and tissues (Bonney et al., 2019). CCM exhibits a high incidence of conduction disorders such as atrial fibrillation and A-V blocks, leading to an increased risk of embolic events and sudden cardiac death, respectively (Bonney et al., 2019; Echeverría et al., 2021). Furthermore, left ventricular aneurysms increase the risk of embolic events such as stroke and mesenteric ischemia (Nunes et al., 2018). Consequently, CCM has one of the highest rates of short-term adverse cardiovascular outcomes across all the etiologies of heart failure (Shen et al., 2017).

Despite its relevance, the pathophysiology of progressive myocardial injury in CCM is still not fully understood, being multiple mechanisms involved in developing the cardiac form of the disease (Bonney and Engman, 2015; Echeverria and Morillo, 2019). One tool that has been used for elucidating the potential pathways involved in several diseases is microRNA analysis. For example, in the setting of heart failure, microRNAs are involved in the genetic regulation process of myocardial function, and their expression has been linked to several cardiovascular disorders (Wang and Cai, 2017). Specifically, studies performed in murine models have highlighted the substantial role of several microRNA expression levels in multiple pathways related to the immune response and *T. cruzi* virulence mechanisms (Linhares-Lacerda et al., 2015; Navarro et al., 2015; Ferreira et al., 2017; Jha et al., 2020; Ballinas-Verdugo et al., 2021).

Similarly, studies performed in human myocardial tissue obtained from transplanted hearts have also reported relevant results, highlighting the study of *Ferreira LRP et al.* in which CCM patients had significantly downregulated levels of miR-1, miR-133a-2, miR-133b, miR-208a, and 208b compared to controls (Ferreira et al., 2014). Furthermore, the study of *Laugier et al.* suggested significantly different expression levels of miR-146a and miR-155 in CCM myocardium samples compared with controls. The pathway analyses of these tissue signatures suggested an enrichment in biological processes associated with the immune response, highlighting inflammation, Th1/IFN-γ-inducible genes, fibrosis, hypertrophy, and mitochondrial/oxidative stress/antioxidant response (Laugier et al., 2020). Nevertheless, up to date, only the study of *Nonaka CKV et al.* has evaluated the association between microRNA expression patterns and clinical variables in CCM patients, observing that miR-19a-3p, miR-21-5p, and miR-29b-3p were differentially expressed in CCM patients compared to those with the indeterminate form of the disease. Furthermore, a positive correlation between the relative expression of these microRNAs with the functional class and fibrosis and a negative correlation with ejection fraction and left ventricular strain was observed. These results were validated by cardiac tissue analysis and *in vitro* studies, suggesting miR-19a-3p, miR-21-5p, and miR-29b-3p circulating levels as potential biomarkers for early detection of disease progression in CD (Nonaka et al., 2019).

Nevertheless, no study has evaluated the potential association between microRNAs’ relative expression and the levels of circulating cardiorenal biomarkers of heart failure severity. Similarly, there is currently no evidence regarding the role of microRNAs in the prognosis of patients with CCM, limiting the possibility of assessing the role of these molecules as clinically useful biomarkers in this setting (Linhares-Lacerda et al., 2018; Ballinas-Verdugo et al., 2021). Considering that multiple studies have observed that miR-34a-5p, miR-208a-5p, miR-185-5p, miR-223-5p, let-7d-5p, and miR-454-5p are significantly associated with HF diagnosis and prognosis, we aimed to analyze the association between the circulating levels of these six microRNAs with markers of myocardial injury and prognosis in CCM patients with a reduced left ventricular ejection fraction (LVEF) (Montgomery et al., 2011; Bernardo et al., 2014; Lv et al., 2014; Yang et al., 2015; Zhang and Schulze, 2016; Peterlin et al., 2020).

## Methods

### Study population

This exploratory prospective cohort study was conducted between 2015 and 2021 at the Heart Failure and Heart Transplant Clinic of Fundación Cardiovascular de Colombia in Floridablanca, Colombia. Adult outpatients (> 18 years old) with a positive serological diagnosis of *T. cruzi* infection (positive IgG antibodies) and echocardiographic or electrocardiographic abnormalities consistent with chronic Chagas cardiomyopathy were included during their follow-up evaluations at the clinic during the year 2015. During this year, samples of whole blood, serum, and plasma of patients enrolled in the study were collected and stored in the biobank of the institution. We enrolled only patients with reduced left ventricular ejection fraction (LVEF), defined as a LVEF ≤ 40%. We excluded individuals with diabetes mellitus, coronary heart disease history, mitral stenosis, or uncontrolled hypertension. The Institutional Committee on Research Ethics approved the research protocol of the study. All patients provided written informed consent for their participation in the study.

### Quantification of human serum levels of microRNA

We used the miRNeasy Serum/Plasma Advanced Kit (Qiagen, Hilden, Germany) to purify total RNA, including small RNAs, from 250 μl of serum following the manufacturer’s instructions. All samples were eluted in 20 μl of RNase/DNase free water. We used a fixed volume of input RNA (14 μl) for the polyadenylation reaction and 10 μl of the polyadenylation reaction for cDNA synthesis following the steps described in the miRNA 1st-Strand cDNA Synthesis Kit (Agilent Technologies, Santa Clara, USA) instruction manual. Quantitative real-time polymerase chain reaction (qRT-PCR) for microRNA quantitation was performed using the miRNA QPCR Master Mix (Agilent Technologies, Santa Clara, USA) on a CFX 96 C1000 Realtime Thermal Cycler (Bio-Rad, Hercules, CA, USA).

The primers used to perform qRT-PCR were the universal reverse primer included in the miRNA 1st-strand cDNA synthesis kit and unique forward primers specific to each of the miRNAs under study. The forward primers were designed to be complementary to the 3´ end of the cDNA strand, were identical in sequence and length to the miRNA itself and were obtained from Macrogen, Korea (**Supplementary Table 1**). Individual qRT-PCR assays were performed in duplicates with a total reaction volume of 12 μL, including the 3 μL of cDNA template. Cycling was set up as follows: 1. 95°C for 10 minutes, 2. 95°C for 10 seconds, 3. 60°C for 15 seconds, 4. 72°C for 20 seconds. Steps 2-4 were repeated 44 times.

We performed absolute quantification of the miRNAs which involve the amplification of templates at known concentrations in order to create a standard curve based on Ct values. For each of the miRNAs under study we generated five-point standard curves from DNA oligonucleotides corresponding to the mature miRNAs. Serial dilutions in nuclease-free water (ranging from 104-108 copies/reaction) of the synthetic oligonucleotides were used as the template for miRNA reverse transcription. All standards and unknown samples were handled in the same manner, and quantitation was performed on the CFX 96 C1000 Realtime Thermal Cycler (Bio-Rad, Hercules, CA, USA) as described above. Ct values from the unknown samples were extrapolated to the standard curve to get the number of copies (**Supplementary Figure 1** and **Supplementary Table 2**).

### Study outcomes and follow-up

After baseline screening, patients were followed up with a telephone interview and provided a standardized checklist of questions to identify clinical outcomes. Further, the clinical records of each patient were revised for additional information and to validate the reported outcomes. The primary outcome was myocardial involvement (assessed by NT-proBNP levels [measured using the electrochemiluminescence method, Roche Diagnostics GmbH, Mannheim, Germany], LVEF, global longitudinal strain value [GLS], LA area, ESV-LV, EDV-LV, LV mass index, TAPSE, Mitral flow E velocity, E/e’ lateral ratio).

The composite outcome (CO) included all-cause mortality, heart transplant, and left ventricular assistance device (LVAD) implantation. Follow-up of each participant began on the date of collection of non-fasting blood samples (2015) and ended at the date of all-cause mortality, heart transplant, LVAD implementation, loss to follow-up, or end of the study period on January 2021, whichever came first.

### Statistical analysis

Categorical variables were presented as numbers and proportions, while continuous variables were reported as medians and interquartile ranges. The Chi-square and Fischer exact tests were used to assess differences in categorical variables. In contrast, the Mann-Whitney U test and the Kruskal-Wallis test were used for continuous variables. Furthermore, we used natural log-transformed values to present the miRNAs’ normalized expression values. The cross-sectional associations of the miRNAs with echocardiographic parameters and NT-proBNP were assessed using multivariable linear regression models adjusted by age and sex (model 1) and age, sex, body mass index, and HF medications (model 2).

Furthermore, we performed survival analyses using the Kaplan-Meier method, life table, and Cox Proportional Hazard models to evaluate the association between the microRNAs relative expression levels and the CO. Similar to the cross-sectional analysis, a basic model (model 1) adjusted by age and sex was constructed. Next, a second model (model 2) adjusting additionally for BMI and HF medications was developed. We assessed the models for non-linear associations using quadratic terms, an incremental F test and the Wald test, graphically representing the observed non-linear associations using the STATA curvefit command. Considering the incidence of the composite outcome, we calculated a statistical power of 83% for observing significant differences equal or larger to 0.7 units in the normalized expression values of the evaluated miRNAs. A p-value <0.05 was considered statistically significant for all tests, but as sensitivity analysis, to account for multiple testing, we adjusted the P-value from 0.05 to 0.005 by applying the Bonferroni correction for the number of exposures studied and taking into account the correlation between the measures. All analyses were performed using Statistical Package STATA version 15 (Station College, Texas, USA).

### Target gene prediction and pathway analysis

The prediction of target genes of the selected miRNA was performed using a strategy described elsewhere (Gupta et al., 2021; Gupta et al., 2021). In brief, three online tools: TargetScan Version 7.2 (http://www.targetscan.org/vert_72/), DIANA-microT-CDS (http://www.microrna.gr/microT-CDS), and MiRDIP (http://ophid.utoronto.ca/mirDIP) were used. We selected the target genes that occurred in at least two databases using Venny2.1.0 (https://bioinfogp.cnb.csic.es/tools/venny/). Finally, pathway enrichment analyses were performed in miRWalk database (v.2.0) and Reactome pathway database Version 81 considering a P<0.05 as the cut-off criterion for selecting relevant pathways after multiple comparisons adjustment by the Benjamini-Hochberg method.

## Results

Seventy-four patients were included, mainly males (59%) with a median age of 64 (Q1: 58, Q3: 72) years at the time of enrolment (**Supplementary Figure 2**). All included patients had a reduced left ventricular ejection fraction (median LVEF 29%, Q1: 21%; Q3: 36%), while the median NT-proBNP value was 2146.5 pg/ul (Q1 = 1021.7; Q3 = 6065.7). Most of the patients were receiving an ACEI/ARB (87.8%), beta-blockers (95.9%), or mineralocorticoid receptor antagonists (82.4%) (**Supplementary Table 3**).

### miRNA levels and CCM severity

**Supplementary Figure 3** summarizes the normalized expression values for the evaluated miRNAs. After adjusting for age and sex in model 1, higher log-natural converted miR-223-5p relative expression levels were associated with a lower left atrium area (Coef. -8.42; 95% CI -15.32; -1.52), lower end-systolic volume (ESV) of the left ventricle (Coef. -45.40; 95% CI -73.78; -17.03), and a lower end-diastolic volume (EDV) of the left ventricle (Coef. -44.86; 95% CI -80.19; -9.53). Furthermore, miR-208a-5p relative expression levels were significantly associated with lower EDV of the left ventricle (Coef. -137.89; 95% CI -275.21; -0.57) and tricuspid annular plane systolic excursion (TAPSE) values (Coef. -8.33; 95% CI (-16.59; -0.06). Finally, higher miR-454-5p levels were associated only with a higher left atrial (LA) area (Coef. 200.36; 95% CI 66.36; 334.35). In model 1, no significant associations were observed for let-7d-5p, miR-185-5p, and miR-34a-5p. Nevertheless, after further adjustment for BMI and HF medications in model 2, only miR-223-5p and miR-454 remained significantly associated with myocardial function markers (**Table 1**). After considering our adjusted p-value derived from the Bonferroni correction (p-value < 0.005), only miR-223-5p remained significantly associated with markers of myocardial involvement (**Table 1**).

**Table 1:** The association between miRNAs levels and markers of myocardial injury in patients with Chronic Chagas Cardiomyopathy (n=74).

Parameter	Models	let-7d-5p* (n=45)		miR-185-5p* (n=50)		miR-208a-5p* (n=32)		miR-223-5p* (n=50)		miR-34a-5p* (n=48)		miR-454-5p* (n=41)	
		β (95% CI)	p-value	β (95% CI)	p-value	β (95% CI)	p-value	β (95% CI)	p-value	β (95% CI)	p-value	β (95% CI)	p-value
NT-proBNP (pg/ml)	Model 1	-527.08 (-3923.70; 2869.54)	0.756	744. 69 (-1924.35; 3413.73)	0.577	-6783.36 (-19422.10; 5855.38)	0.278	-522 (-3626.81; 2582.49)	0.736	765.33 (-4130.48; 5661.14)	0.754	-13419.77 (-79328.32; 52488.77)	0.682
	Model 2	983.34 (-2276.32; 4242.99)	0.545	-88.12 (-2549.95; 2373.70)	0.943	-2894.65 (-8194.43; 2405.13)	0.268	-48.72 (-3086.83; 2989.38)	0.974	-743.99 (-4747.21; 3258.22)	0.709	-4046.96 (-67333.6; 59239.7)	0.897
LVEF (%)	Model 1	2.20 (-1.48; 5.88)	0.234	-0.15 (-4.52; 4.21)	0.944	10.39 (-5.10; 25.88)	0.179	3.03 (-1.08; 7.13)	0.144	0.11 (-5.27; 5.48)	0.969	-2.41 (-6.11; 1.28)	0.194
	Model 2	1.30 (-2.72; 5.32)	0.516	-0.46 (-4.93; 4.01)	0.835	13.18 (-3.16; 29.53)	0.108	3.58 (-0.39; 7.55)	0.076	-0.71 (-6.41; 4.99)	0.802	-79.97 (-183.19; 23.24)	0.124
GLS (%)	Model 1	-1.06 (-2.55; 0.43)	0.158	-0.22 (-2.15; 1.71)	0.821	-1.83 (-7.69; 4.03)	0.522	-1.08 (-2.82; 0.66)	0.219	-0.19 (-2.37; 1.98)	0.860	2.31 (-42.75; 47.38)	0.917
	Model 2	-0.86 (-2.49; 0.78)	0.296	0.01 (-1.91; 1.93)	0.993	-2.93 (-9.16; 3.29)	0.337	-1.34 (-3.06; 0.39)	0.126	0.61 (-1.61; 2.83)	0.579	10.14 (-34.28; 54.56)	0.644
LA Area (cm2)	Model 1	0.36 (-5.69; 6.41)	0.903	-1.52 (-11.59; 8.64)	0.758	-37.97 (-82.92; 6.98)	0.089	**-8.42 (-15.32; -1.52)**	**0.019**	1.88 (-6.56; 10.33)	0.649	200.36 (66.36; 334.35)	**0.005**
	Model 2	-3.74 (-9.87; 2.39)	0.218	-2.56 (-12.82; 7.68)	0.609	-24.89 (-89.17; 39.38)	0.398	**-10.21 (-16.35; -4.09)**	**0.002**	-0.65 (-10.19; 8.90)	0.889	168.33 (4.35; 332.29)	**0.045**
ESV-LV (ml)	Model 1	-12.85 (-39.03; 13.33)	0.322	-25.62 (-73.86; 22.62)	0.284	-110.38 (-246.81; 26.04)	0.102	**-45.40 (-73.78; -17.03)**	**0.003**	1.24 (-39.55; 42.02)	0.950	466.35 (-418.15; 1350.86)	0.284
	Model 2	-11.65 (-42.94; 19.65)	0.448	-23.65 (-76.31; 29.01)	0.361	-161.91 (-352.21; 28.39)	0.085	**-45.34 (-74.06; -16.61)**	**0.004**	3.29 (-45.76; 52.36)	0.889	726 (-462.64; 1914.68)	0.214
EDV-LV (ml)	Model 1	-11.26 (-41.19; 18.68)	0.446	-23.68 (-77.78; 30.43)	0.376	***-137.89 (-275.21; -0.57)***	***0.049***	**-44.86 (-80.19; -9.53)**	**0.015**	4.32 (-39.37; 48.00)	0.840	619.26 (-375.59; 1614.12)	0.209
	Model 2	-9.50 (-44.59; 25.58)	0.579	-19.72 (-77.53; 38.10)	0.486	-187.01 (-380.47; 6.46)	0.056	**-46.13 (-81.99; -10,26)**	**0.014**	5.54 (-46.45; 57.53)	0.825	900.61 (-440.06; 2241.28)	0.174
LV mass index (g/m2)	Model 1	9.99 (-14.29; 34.26)	0.411	11.22 (-14.65; 37.09)	0.387	-74.39 (-171.38; 22.61)	0.126	-1.22 (-29.24; 26.79)	0.930	26.06 (-7.14; 59.26)	0.121	-23.01 (-631.90; 585.89)	0.939
	Model 2	13.72 (-13.06; 40.49)	0.306	7.99 (-19.71; 35.71)	0.563	-68.88 (-180.82; 43.05)	0.214	1.70 (-28.35; 31.75)	0.909	20.61 (-16.62; 57.82)	0.270	-9.16 (-672.68; 654.25)	0.978
TAPSE (mm)	Model 1	-1.24 (-3.59; 1.11)	0.293	-0.19 (-2.44; 2.40)	0.987	***-8.33 (-16.59; -0.06)***	***0.048***	1.23 (-1.36; 3.83)	0.345	-0.49 (-3.59; 2.61)	0.751	28.85 (-33.12; 90.81)	0.352
	Model 2	-1.43 (-3.91; 1.05)	0.251	0.38 (-2.09; 2.86)	0.757	-8.01 (-17.18; 1.17)	0.084	0.59 (-2.09; 3.28)	0.659	-1.49 (-4.80; 1.81)	0.365	34.15 (-32.08; 100.39)	0.302
Mitral flow E velocity (cm/s)	Model 1	-3.44 (-17.02; 10.14)	0.611	1.03 (-14.76; 16.82)	0.896	22.98 (-36.61; 82.56)	0.433	4.86 (-14.21; 23.94)	0.610	1.38 (-16.97; 19.74)	0.880	343.99 (-42.87; 730.86)	0.080
	Model 2	-6.97 (-21.49;7.55)	0.337	0.91 (-14.88; 16.71)	0.907	15.09 (-45.11; 75.29)	0.607	4.11 (-15.72; 23.93)	0.678	6.74 (-13.01; 26.49)	0.493	347.25 (-92.14; 786.63)	0.117
E/e’ lateral ratio	Model 1	0.03 (-2.19; 2.25)	0.980	-0.02 (-2.87; 2.82)	0.986	-4.22 (-14.20; 5.76)	0.387	0.77 (-2.37; 3.91)	0.622	-0.09 (-3.01; 2.82)	0.950	9.87 (-41.77; 61.50)	0.699
	Model 2	-0.32 (-2.87; 2.22)	0.796	-0.39 (-3.12; 2.34)	0.773	-2.93 (-12.57; 6.70)	0.530	0.50 (-2.77; 3.77)	0.757	0.34 (-2.73; 3.41)	0.823	2.64 (-57.42; 62.69)	0.929

*Natural log-transformed values.

Bold text represent p-values < 0.05.

Model 1: Model adjusted by age and sex.

Model 2: Model adjusted by sex, age, BMI, and HF medications.

Considering the trends observed for miR-223-5p and the study’s small sample size, we compared the mean levels of this miRNA according to the NT-proBNP levels, the LVEF, and GLS divided into two groups (over the median value and below the median value). We observed that patients with an NT-proBNP value > 2150 pg/ul had a significantly lower hsa-miR-223-5p relative expression. A similar result was observed for the LVEF, highlighting that patients with an ejection fraction >29% had a significantly higher miR-223-5p relative expression. Finally, we did not observe a significant association for the GLS.

### Impact of microRNAs’ relative expression levels on the composite outcome

During the median follow-up of 40 months (Q1: 23; Q3: 52), 43% of participants reported an event of the CO, with a rate of 0.40 per 1000 person-years (95% CI 0.28-0.56). The Cox proportional hazard models suggested that none of the evaluated microRNAs was significantly associated with the composite outcome (**Supplementary Table 4**).

### Sensitivity analysis

No significant interaction terms by sex were observed in any of the analyses. Similarly, we evaluated the possibility of non-linear associations between the microRNAs and the assessed variables, observing a significant quadratic term for miRNA-223-5p association with the left atrium area (Change in the R^2^: 0.12. p-value=0.044) (**Supplementary Figure 4**). No other significant associations regarding non-linear trends were observed.

### Predicted miRNA targets

We further performed a bioinformatic analysis on miR-223-5p, as it was the microRNA shown to be the most significantly associated with the evaluated outcomes. We observed a total of 123 potential target genes identified in the analysis, being eight present in the three queried databases, and the rest in two of them (**Supplementary Table 5**).

### Functional and pathway analysis

**Figure 1:**
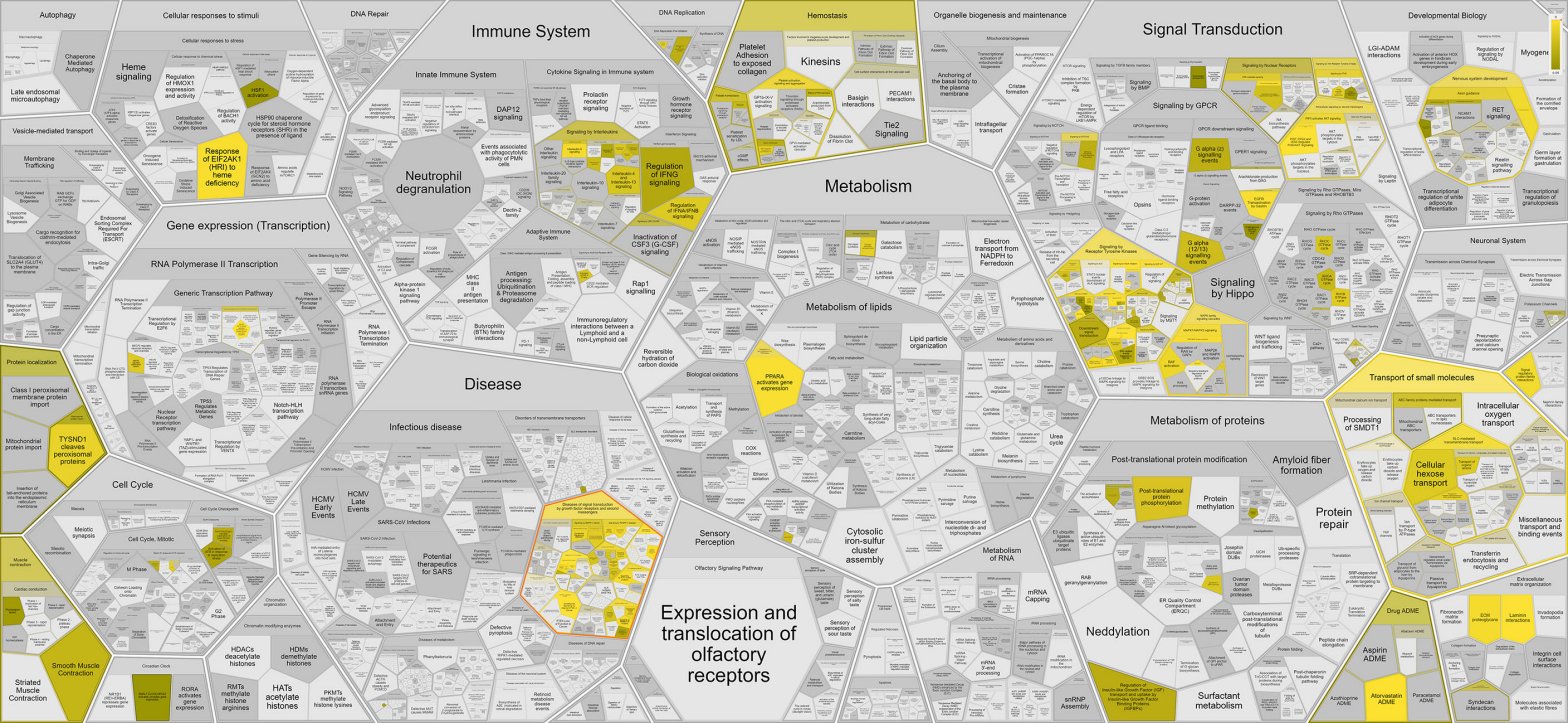
Graphical representation of the main Reactome pathways enriched in the target genes from hsa-miR-223-5p. The intensity of the color is related to the statistical significance of the miRNA involvement, while the cell size is related to the number of genes in each pathway.

To elucidate the pathways potentially associated with disease severity in CCM related to miR-223-5p expression, the target genes were subjected to gene set enrichment analysis using miRWalk. The genes were observed to be involved in several biological processes, highlighting specific pathways related to signaling by receptor tyrosine kinases such as MAPK signaling pathway (hsa04010) and mTOR signaling pathway (hsa04150) (**Tables 2**, **3**, and **Figure 1**).

**Table 2:** Gene set enrichment analysis of target genes for hsa- miR-223-5p.

Name	Hits	Pop Hits	List Total	Genes	Pvalue	Adjusted P-value
Gene Ontology						
GO:0006699_bile_acid_biosynthetic_process	5	48	106	18160	0.0	0.0001
GO:0008284_positive_regulation_of_cell_population_proliferation	13	584	106	18160	0.0001	0.0006
GO:1902476_chloride_transmembrane_transport	6	114	106	18160	0.0001	0.0006
GO:0006303_double-strand_break_repair_via_nonhomologous_end_joining	5	96	106	18160	0.0004	0.0019
GO:0006874_cellular_calcium_ion_homeostasis	5	115	106	18160	0.0008	0.003
GO:0008543_fibroblast_growth_factor_receptor_signaling_pathway	5	133	106	18160	0.0015	0.0048
GO:0045893_positive_regulation_of_transcription,_DNA-templated	12	752	106	18160	0.0028	0.0076
GO:0030154_cell_differentiation	10	611	106	18160	0.0049	0.0116
GO:0005925_focal_adhesion	10	498	77	12816	0.0016	0.0144
GO:0034220_ion_transmembrane_transport	5	211	106	18160	0.0098	0.0207
GO:0001228_DNA-binding_transcription_activator_activity,_RNA_polymerase_II-specific	10	500	95	15238	0.002	0.0222
GO:0005125_cytokine_activity	6	222	95	15238	0.0037	0.0222
GO:0004674_protein_serinethreonine_kinase_activity	10	609	95	15238	0.0076	0.0304
GO:0007411_axon_guidance	5	246	106	18160	0.0176	0.0334
GO:0007268_chemical_synaptic_transmission	5	259	106	18160	0.0214	0.037
GO:0003677_DNA_binding	13	984	95	15238	0.0143	0.0382
GO:0003924_GTPase_activity	7	412	95	15238	0.0191	0.0382
GO:0008134_transcription_factor_binding	6	310	95	15238	0.0165	0.0382
GO:0003682_chromatin_binding	7	439	95	15238	0.0256	0.0384
GO:0005525_GTP_binding	7	427	95	15238	0.0225	0.0384
GO:0006915_apoptotic_process	8	571	106	18160	0.0244	0.0386
**KEGG**						
hsa04010_MAPK_signaling_pathway	7	295	52	8019	0.0051	0.0153
hsa04150_mTOR_signaling_pathway	5	155	52	8019	0.0048	0.0153
hsa05205_Proteoglycans_in_cancer	5	204	52	8019	0.0141	0.0282
hsa04014_Ras_signaling_pathway	5	232	52	8019	0.0228	0.0342

**Table 3:** Reactome pathway analysis of target genes for hsa- miR-223-5p.

Pathway name	Entities found	Entities total	Entities ratio	p-value	Adjusted p-value
Negative regulation of the PI3K/AKT network	12	137	0.009	0.0000132	0.00396
Signaling by Receptor Tyrosine Kinases	28	620	0.041	0.0000177	0.00396
PI5P, PP2A and IER3 Regulate PI3K/AKT Signaling	11	129	0.009	0.0000385	0.00675
SHC-mediated cascade:FGFR2	6	33	0.002	0.0000425	0.00675
FRS-mediated FGFR2 signaling	6	34	0.002	0.00005	0.00683
Extra-nuclear estrogen signaling	10	111	0.007	0.000055	0.00683
Insulin receptor signalling cascade	8	72	0.005	0.0000741	0.00823
Downstream signaling of activated FGFR2	6	42	0.003	0.000157	0.0135
IRS-mediated signalling	7	65	0.004	0.000251	0.0173
Laminin interactions	5	31	0.002	0.000322	0.0208
Negative regulation of MAPK pathway	6	49	0.003	0.000355	0.0208
IRS-related events triggered by IGF1R	7	69	0.005	0.000359	0.0208
IGF1R signaling cascade	7	72	0.005	0.000461	0.0254
Signaling by Type 1 Insulin-like Growth Factor 1 Receptor (IGF1R)	7	73	0.005	0.0005	0.0265
Signaling by Insulin receptor	8	97	0.006	0.000541	0.0271
Atorvastatin ADME	4	20	0.001	0.000586	0.0278
SHC1 events in ERBB2 signaling	5	36	0.002	0.000632	0.0278
TFAP2 (AP-2) family regulates transcription of growth factors and their receptors	4	21	0.001	0.000702	0.0302

## Discussion

Despite significant advances in the study of miRNAs as new biomarkers and therapeutic targets in the area of acute myocardial infarction and heart failure, lesser advances have been achieved in the setting of Chagas Disease and Chronic Chagas Cardiomyopathy (Wang and Cai, 2017; Peterlin et al., 2020). Our study results suggested that miR-223-5p expression is significantly associated with disease severity in CCM patients, independent of age and sex, with higher relative expression levels of this miRNA reflected in better myocardial function markers. Furthermore, our pathway enrichment analysis indicated that the role of this miRNA could be related to pathways involved in the adaptive immune system, specifically related to signaling by receptor tyrosine kinases. Our exploratory study provides new insights into the potential role of microRNAs in CD and CCM pathophysiology.

Several microRNAs have been reported in the literature to be involved in the pathophysiology and progression of CD. For example, the study of *Jha BK et al.* observed that miR-155 knockout (KO) mice had a significantly worse prognosis after *T. cruzi* infection compared with wild-type C57BL/6 mice. They found that, in the absence of this microRNA, a decreased release of tumor necrosis factor-alpha (TNF-α) and interferon-gamma (IFN-y) was observed. At the same time, there was an increased accumulation of neutrophils and inflammatory monocytes and a reduced amount of natural killer cells and CD8-positive T cells in the involved tissue. These results suggested a critical role of miR-155 in the immune regulation in response to *T. cruzi* infection (Jha et al., 2020). Similar to this, additional studies on murine models have observed a substantial role of several microRNA expression levels in multiple pathways related to the immune response and *T. cruzi* virulence mechanisms (Navarro et al., 2015; Linhares-Lacerda et al., 2015; Ferreira et al., 2017; Ballinas-Verdugo et al., 2021).

Similarly, studies performed in human myocardial tissue obtained from transplanted hearts have also reported relevant results, highlighting the study of *Ferreira LRP et al.* In this study, the authors analyzed the expression of nine microRNAs in myocardial tissue samples of three populations: CCM patients, dilated cardiomyopathy patients and healthy controls, observing that miR-1, miR-133a-2, miR-133b, miR-208a, and 208b were significantly down-regulated in CCM patients compared to controls. The pathway enrichment analysis suggested that the target genes for these microRNAs were involved in the network labeled as “Cardiovascular Disease, Connective Tissue Disorders, Dermatological Diseases” (Ferreira et al., 2014). Furthermore, the study of *Laugier et al.* suggested significantly different expression levels of miR-146a and miR-155 in CCM myocardium samples compared with controls. The pathway analyses of these tissue signatures suggested an enrichment in biological processes associated with the immune response, highlighting inflammation, Th1/IFN-γ-inducible genes, fibrosis, hypertrophy, and mitochondrial/oxidative stress/antioxidant response (Laugier et al., 2020). Nevertheless, up to date, only the study of *Nonaka CKV et al.* has evaluated the association between microRNA expression patterns and clinical variables in CCM patients, assessing six circulating microRNAs (miR-19a-3p, miR-21-5p, miR-29b-3p, miR-30a-5p, miR-199b-5p, and miR-208a-3p). In this study, the authors observed that miR-19a-3p, miR-21-5p, and miR-29b-3p were differentially expressed in CCM patients compared to those with the indeterminate form of the disease. Furthermore, a positive correlation between the relative expression of these microRNAs with functional class and fibrosis and a negative correlation with ejection fraction and left ventricular strain was observed. These results were validated by cardiac tissue analysis and *in vitro* studies, suggesting miR-19a-3p, miR-21-5p, and miR-29b-3p circulating levels as potential biomarkers for early detection of disease progression in CD (Nonaka et al., 2019).

While microRNAs have been previously suggested to play a role in Chagas disease, our study represents the first to investigate and observe the significant role of miR-223-5p in the pathophysiology of CD and CCM. MiR-223, a microRNA expressed in multiple cells such as platelets, macrophages, and cardiomyocytes, has been observed to be involved in several cardiovascular diseases. Nevertheless, most of the available evidence has evaluated the 3’-arm of precursor miR-223, as it was considered the only mature and functional component, while the complementary 5’-arm was thought to be degraded (Mitra et al., 2015; Córdova-Rivas et al., 2019). Recent evidence has suggested a co-expression of both arms of pre-miR-223 in relevant pathophysiological processes affecting the myocardium (Taïbi et al., 2014; Wang et al., 2014). In the study of *Wang et al.*, the knockout (KO) of the miR-223 duplex (5p and 3p) promoted a most severe sepsis-induced cardiac dysfunction and higher mortality in KO mice compared with wild-type ones (Wang et al., 2014). A similar result was observed by *Qin et al.* in the setting of myocardial ischemia/reperfusion-induced necrosis in mice (Qin et al., 2016). In this study, miR-223-5p KO mice hearts exhibited worse recovery of contractile performance over the reperfusion period and a higher degree of necrosis than wild-type ones. The authors suggested the activation of the RIP1/RIP3/MLKL necroptotic pathway as responsible for the differences observed in both groups, as treatment of KO mice with necrostatin-1s, a RIP1 kinase activity suppressor and necroptosis inhibitor, decreased the infarction size and myocardial dysfunction after ischemia/reperfusion injury (Qin et al., 2016). Interestingly, regulated necrosis, or necroptosis, has been observed to be mediated by tumor necrosis factor-α (TNF-α)/TNFR1-induced protein complex RIP1-RIP3, also known as the necrosome (Silke et al., 2015). In this context, several studies have suggested that the TNF-α/TNFR-mediated pathway plays a critical role in the process of *T. cruzi* control in the acute stage and the development of CCM, being TNFR1 closely linked to CD8+ T cell dysregulation (Michailowsky et al., 2001; Tzelepis et al., 2007). Nonetheless, although necroptosis have been studied in heart failure as one of the potential drivers of myocardial remodeling, no study has assessed the role of this mechanism in CD or CCM (Li et al., 2014; Guo et al., 2017).

Finally, we also observed a trend concerning the expression of miR-208a-5p and markers of myocardial function such as the ESV and the TAPSE. Nevertheless, the associations had a borderline statistical significance, and no other trend was observed for the other biomarkers. Therefore, we did not include this microRNA in our bioinformatics analysis. In contrast to miR-223-5p, miR-208a-3p has been previously studied in CD and CCM, highlighting the study of *Linhares-Lacerda L et al.*, which observed significantly higher circulating levels plasma miR-208a in patients with the indeterminate form of the disease than in controls (Linhares-Lacerda et al., 2018). On the other hand, the study of *Ferreira LRP et al.* observed that patients with CCM had a significantly lower relative expression of miR-208a, measured in human myocardial samples, than healthy controls (Ferreira et al., 2014). MiR-208a represents an essential regulator of several pathways involved in cardiac fibrosis and hypertrophy, being also associated with the development of conduction abnormalities and arrhythmias (Wang et al., 2014; Huang et al., 2021). Although the role of this microRNA in CD is not clear, published evidence suggests that TGF-β may activate miR-208a to regulate genes related to hypertrophy, as TGF-β neutralizing antibody therapy attenuated miR-208a induced expression in myocytes (Wang et al., 2013). In this context, the TGF-β signaling pathway has been shown to play a significant role in the development of CCM, as it may potentiate the parasitic burden by downregulating the intracellular immune response against *T. cruzi (* Ferreira et al., 2016; da et al., 2019). Moreover, the study of *Araújo et al.* reported that circulating levels of TGF-β were significantly associated with cardiac dysfunction and fibrosis in CD (Araújo-Jorge et al., 2002), while the study of *Ferreira RR et al.* observed a positive therapeutic effect in mice infected with *T. cruzi* and treated with a selective inhibitor of TβR1/ALK5 (Ferreira et al., 2019), highlighting the relevance of this cytokine in this setting and the potential of miR-208a as a critical mediator of this effect.

## Study strengths and limitations

The present study has relevant strengths worthy of mention, highlighting the inclusion of a population of patients with advanced-stage heart failure and a relatively long follow-up. On the other hand, several limitations are present. First, our study has a small sample size and limited events for the composite outcomes and therefore may not have had enough power, especially for the CO. Second, the cross-sectional design of the study does not provide insights into the causality of the identified associations. Third, the measurement of the microRNAs also carried significant processing issues, highlighting the lack of pre-amplification of serum RNAs due to limited resources, the wide variability of results observed and the low concentrations of these microRNAs in serum. Furthermore, microRNA measurement was performed using a single serum sample, limiting the possibilities of assessing changes over time. Finally, it was not possible to perform an experimental validation of the potential target genes, which limited the possibility of confirming the findings of the prediction analysis performed.

## Conclusions

Our exploratory study observed a significant association between the expression levels of circulating miR-223-5p and echocardiographic and laboratory biomarkers of myocardial injury in patients with CCM. Furthermore, the bioinformatics analysis suggested an important role of this microRNA in pathways related to signaling by receptor tyrosine kinases. Further large prospective cohort studies are required to potentially validate this and other relevant microRNAs as biomarkers or therapeutic targets in the setting of CD and CCM.

## Data availability statement

The raw data supporting the conclusions of this article will be made available by the authors upon request, without undue reservation.

## Ethics statement

The studies involving human participants were reviewed and approved by Comité de etica de la Fundación Cardiovascular de Colombia. The patients/participants provided their written informed consent to participate in this study.

## Author contributions

SG-O, LR, LH, TM, and LE contributed to the conception and design of the study. PB-N did the microRNA extraction and measurements. SG-O, PB-N, and LR organized the database. SG-O, LR, and TM performed the statistical analysis. SG-O wrote the first draft of the manuscript. SG-O, LH, and TM wrote sections of the manuscript. All authors contributed to manuscript revision, read, and approved the submitted version.

## Funding

LR and TM were supported by St. Gallen University through the Seed Money Grants Call of 2019.

## Conflict of interest

The authors declare that the research was conducted in the absence of any commercial or financial relationships that could be construed as a potential conflict of interest.

## Publisher’s note

All claims expressed in this article are solely those of the authors and do not necessarily represent those of their affiliated organizations, or those of the publisher, the editors and the reviewers. Any product that may be evaluated in this article, or claim that may be made by its manufacturer, is not guaranteed or endorsed by the publisher.
