# Supplementary material for: Circulating MicroRNAs and myocardial involvement severity in chronic Chagas cardiomyopathy
Source: Front Cell Infect Microbiol. 2022 Aug 8;12:922189. doi: 10.3389/fcimb.2022.922189 (PMC9393411; doi:10.3389/fcimb.2022.922189)
Supplement: Supplementary file 1 [file DataSheet_1.docx]

**Circulating MicroRNAs and Myocardial Involvement Severity in Chronic Chagas Cardiomyopathy**

**Supplementary Material**

**Supplementary Table 1.** Forward primer sequences.

| **miRNA** | **Sequence** |
| --- | --- |
| hsa-miR-34a-5p | 5’-TGGCAGTGTCTTAGCTGGTTGT-3’ |
| hsa-miR-208a-5p | 5’-GAGCTTTTGGCCCGGGTTATAC-3’ |
| hsa-miR-223-5p | 5’-CGTGTATTTGACAAGCTGAGTT-3’ |
| hsa-miR-185-5p | 5’-TGGAGAGAAAGGCAGTTCCTGA-3’ |
| hsa-miR-7d-5p | 5’-AGAGGTAGTAGGTTGCATAGTT-3’ |
| hsa-miR-454-5p | 5’-ACCCTATCAATATTGTCTCTGC-3’ |

**Supplementary Table 2.** qPCR Efficiency for each evaluated microRNA.

| **miRNA** | **R2** | **Slope** | **qPCR Efficiency (%)** |
| --- | --- | --- | --- |
| 34a-5p | 0,996 | -3,237 | 103,6 |
| 208-5p | 0,989 | -3,723 | 85,6 |
| 223-5p | 0,997 | -3,466 | 94,3 |
| 185-5p | 0,995 | -3,412 | 96,4 |
| 7d-5p | 0,994 | -2,45 | 155,9 |
| 454-5p | 0,993 | -3,056 | 112,4 |

**Supplementary Table 3.** Baseline characteristics of the cohort of patients with chronic Chagas cardiomyopathy.

| **Variables** | **Total (N=74)** |
| --- | --- |
| Males | 44 (59.5%) |
| Age | 64.0 (58.0, 71.5) |
| BMI | 23.5 (21.5, 27.4) |
| NYHA |  |
| I-II | 57 (79.2%) |
| III-IV | 15 (20.8%) |
| ACEI/ARB | 65 (87.8%) |
| Beta-blockers | 71 (95.9%) |
| MRA | 61 (82.4%) |
| Diuretics | 46 (62.2%) |
| Digitalis | 19 (25.7%) |
| Ivabradine | 2 (2.7%) |
| Antiplatelets | 14 (18.9%) |
| Anticoagulants | 39 (52.7%) |
| NT-proBNP (pg/ml) | 2146.5 (1021.7, 6065.7) |
| DHEA-S (µg/dL) | 58.7 (35.8, 1.2) |
| LVEF (%) | 29.0 (21.0, 36.0) |
| GLS (%) | -7.8 (-10.6, -5.8) |
| ESV-LV (ml) | 107.0 (76.0, 142.0) |
| EDV-LV (ml) | 158.0 (121.0, 192.0) |
| LV mass index (g/m2) | 146.8 (117.4, 184.2) |
| TAPSE (mm) | 13.5 (10.8, 17.0) |
| Mitral flow E velocity (cm/s) | 73.0 (50.0, 93.0) |
| E/e' lateral ratio | 9.8 (7.9, 13.4) |
| LA volume index (mL/m2) | 61.0 (45.0, 77.0) |

**Supplementary Table 4.** Association of microRNAs relative expression levels and the composite outcome in patients with chronic Chagas cardiomyopathy and reduced ejection fraction (N = 74).

| **Composite outcome** | **let-7d-5p*** | | **miR-185-5p*** | | **miR-208a-5p*** | | **miR-223-5p*** | | **miR-34a-5p*** | | **miR-454-5p*** | | |
| --- | --- | --- | --- | --- | --- | --- | --- | --- | --- | --- | --- | --- | --- |
|  | *β (95% CI)* | *p-value* | *β (95% CI)* | *p-value* | *β (95% CI)* | *p-value* | *β (95% CI)* | *p-value* | *β (95% CI)* | *p-value* | *β (95% CI)* | *p-value* |  |
| Unadjusted model | 1.17 (0.56; 2.47) | 0.676 | 0.69 (0.31; 1.55) | 0.368 | 1.92 (0.15; 25.03) | 0.617 | 0.96 (0.41; 2.25) | 0.930 | 1.36 (0.53; 3.48) | 0.525 | 0.21 (0.12; 0.34) | 0.868 |  |
| Model 1 | 1.18 (0.56; 2.52) | 0.664 | 0.69 (0.31; 1.55) | 0.368 | 1.79 (0.11; 28.75) | 0.680 | 0.99 (0.43; 2.31) | 0.994 | 1.37 (0.54; 3.49) | 0.512 | 0.09 (0.01; 0.42) | 0.815 |  |
| Model 2 | 1.44 (0.67; 3.08) | 0.347 | 0.68 (0.29; 1.58) | 0.370 | 1.06 (0.07; 16.36) | 0.964 | 0.93 (0.39; 2.21) | 0.875 | 1.77 (0.56; 5.59) | 0.332 | 0.10 (0.01; 0.63) | 0.503 |  |

**Supplementary Table 5.** Target genes prediction for hsa-miR-223-5p.

| **Official Gene Symbol** |
| --- |
| **Present in the three databases** |
| CHAC2 |
| FGF7 |
| PMP2 |
| ASF1A |
| CHIC1 |
| TMEM209 |
| UBE2V2 |
| ETV1 |
| ATP2C1 |
| **Present in two databases** |
| GABRB3 |
| BACH2 |
| PPARGC1B |
| ZC3H12B |
| PBRM1 |
| ERG |
| TOMM20 |
| REV3L |
| NUS1 |
| HMX2 |
| SLC24A2 |
| GLRA2 |
| DIP2B |
| TMEM220 |
| LIFR |
| ZFR |
| FOXC1 |
| SPOPL |
| ALDH1L2 |
| GTPBP2 |
| RGS7BP |
| MMP16 |
| ZNF441 |
| LPP |
| EFNB2 |
| AKTIP |
| XPO4 |
| ZBTB10 |
| GPM6A |
| MAP3K2 |
| KCNJ13 |
| RPS6KA3 |
| AIMP1 |
| RAB22A |
| MAB21L1 |
| RMND5A |
| DYNLT1 |
| ZFPM2 |
| SRSF2 |
| POU3F2 |
| LRP6 |
| DLC1 |
| ZZZ3 |
| BCL11A |
| NHSL1 |
| PLA2G12A |
| WDR35 |
| TSLP |
| FNDC3B |
| GOLGA1 |
| SNX27 |
| ARHGAP5 |
| CYP7A1 |
| EFHC1 |
| SEMA3A |
| INO80D |
| IGF1 |
| C10orf90 |
| TMEM170B |
| XRCC5 |
| AFTPH |
| SCML4 |
| ENTPD1 |
| ROCK2 |
| RRAS2 |
| PCDH11Y |
| KLHL9 |
| TFAP2B |
| ARF1 |
| UBE2Q2 |
| APBB2 |
| EPB41L4B |
| TAOK1 |
| SMAD2 |
| SLC44A1 |
| SLC2A1 |
| FGF12 |
| ARID4B |
| YIPF6 |
| KMT2C |
| NAALADL2 |
| PTGES3 |
| RBM39 |
| PALLD |
| DMD |
| ZNF644 |
| PRDM4 |
| RBMS1 |
| PPP6R3 |
| DDIT4 |
| ANTXR1 |
| HTR5A-AS1 |
| LINC00955 |
| AL138847.1 |
| ARL9 |
| DEFB110 |
| KRTAP13-1 |
| DENR |
| KRBOX1 |
| TEX30 |
| RP11-17M16.1 |
| DLK2 |
| TRAPPC3L |
| PMS2 |
| OSTN |
| SPACA1 |
| GRB2 |
| TNFSF14 |
| SETD9 |
| AKAP4 |
| AC015987.2 |
| TNFSF10 |
| AC024940.1 |


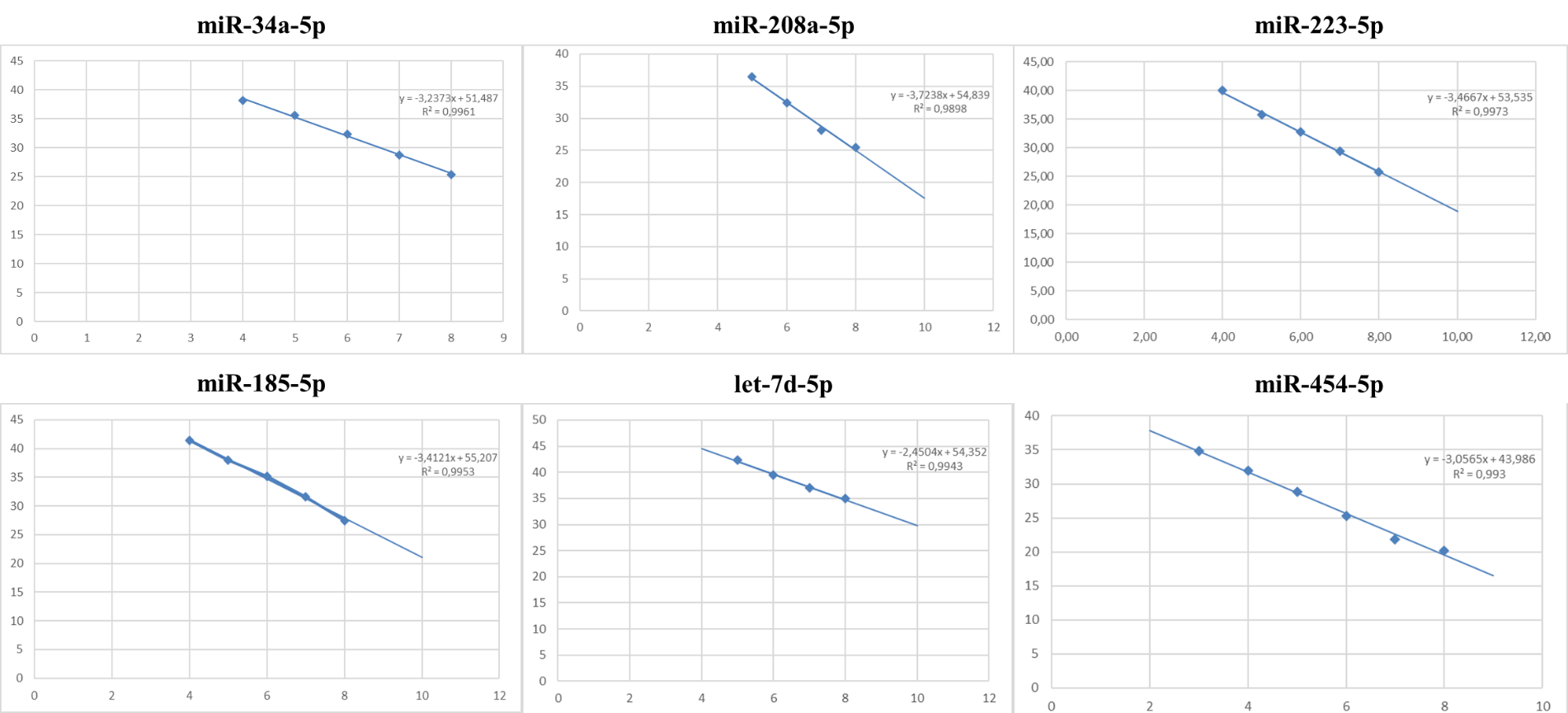


**Supplementary Figure 1.** Standard curves and formulas for extrapolating the number of copies of each miRNA.


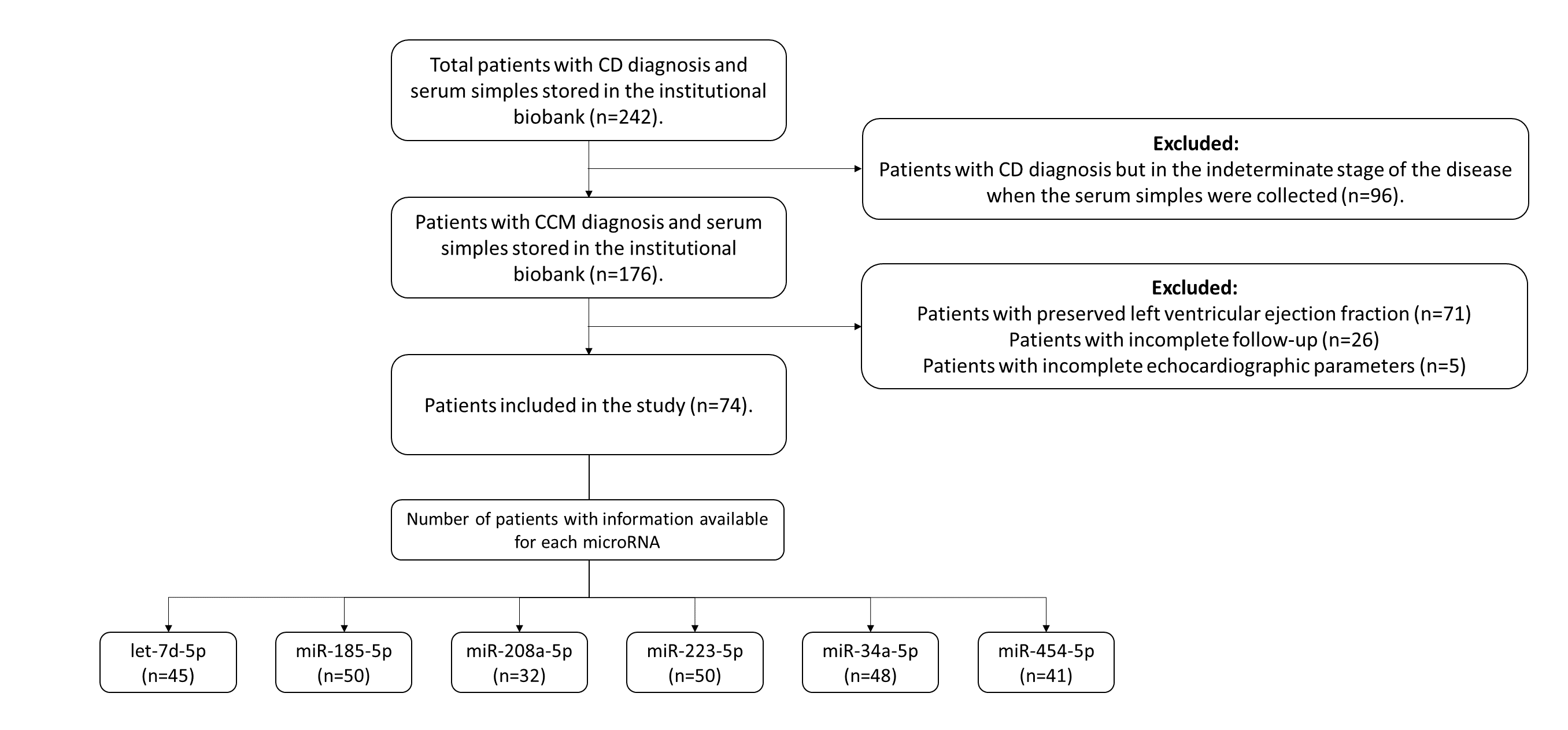


**Supplementary Figure 2.** Flowchart summarizing the selection process of the assessed cohort of patients.


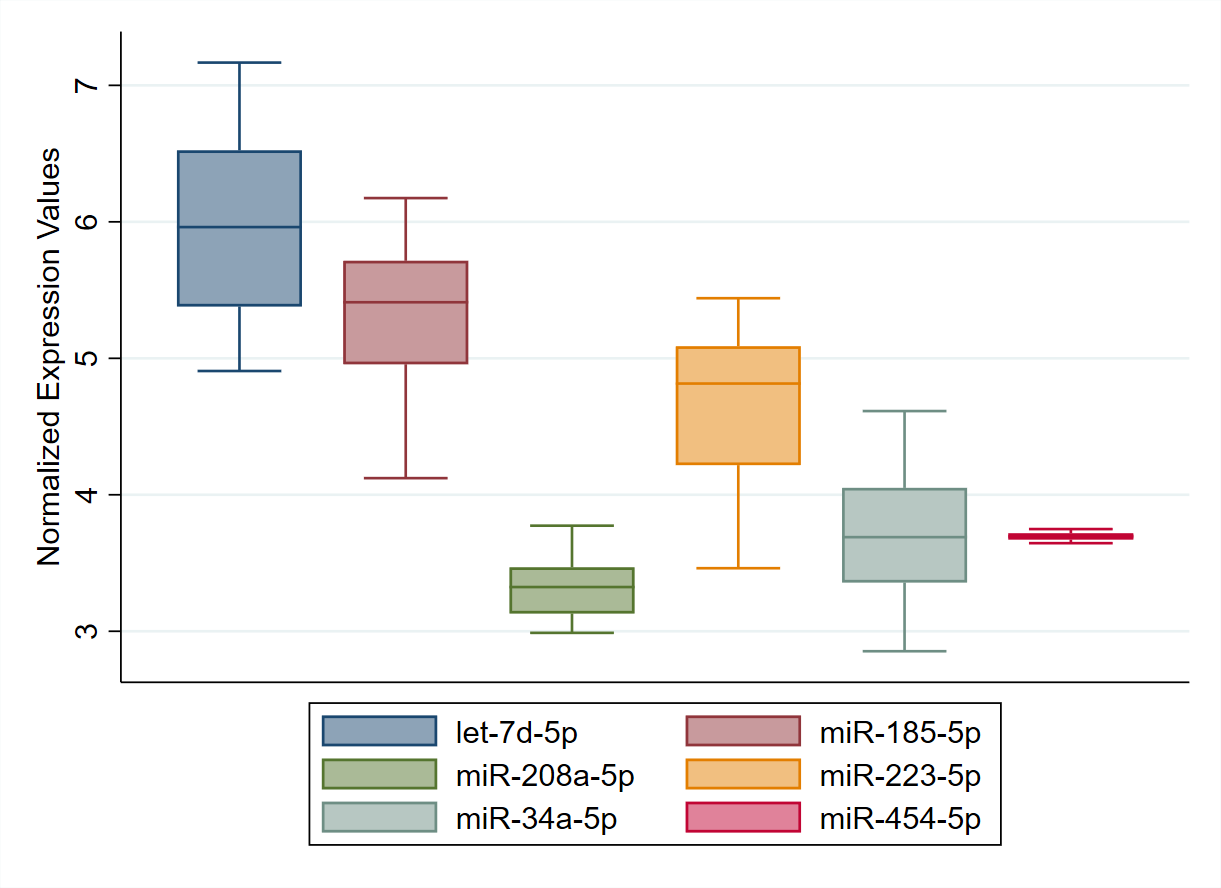


**Supplementary Figure 3.** Expression profiling of six microRNAs in serum samples from patients with Chronic Chagas Cardiomyopathy.


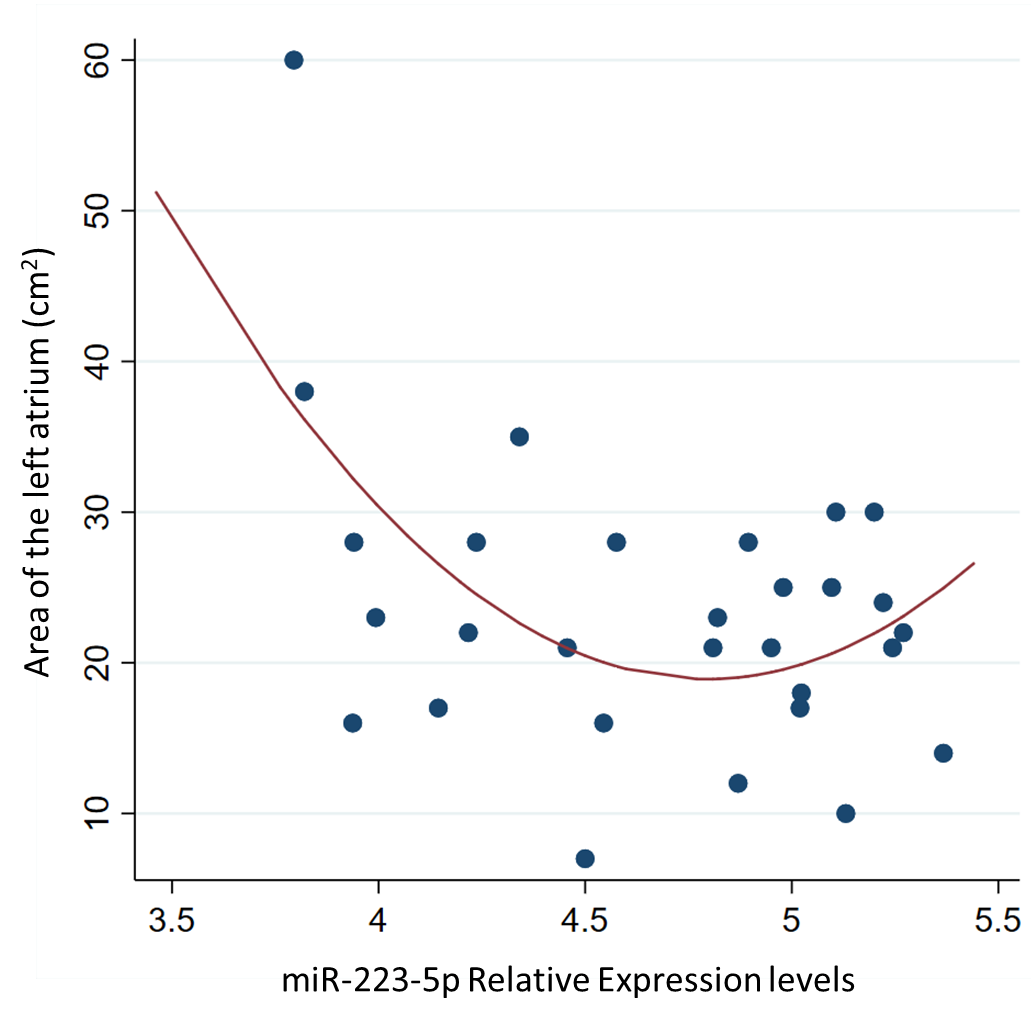


**Supplementary Figure 4.** Scatter plot depicting the association between left atrial area and relative miR-223-5p expression levels. We highlight the better fit of a quadratic distribution (R^2^= 0.22) rather than a linear one (R^2^= 0.12).
